# Fetal growth does not modify the relationship of infant weight gain with childhood adiposity and blood pressure in the Southampton women’s survey

**DOI:** 10.1080/03014460.2020.1717616

**Published:** 2020-05-20

**Authors:** Tom Norris, Sarah R. Crozier, Noël Cameron, Keith M. Godfrey, Hazel Inskip, William Johnson

**Affiliations:** a School of Sport, Exercise and Health Sciences, Loughborough University, Loughborough, UK;; b MRC Lifecourse Epidemiology Unit, University of Southampton, Southampton, UK

**Keywords:** Estimated fetal weight, infant weight gain, childhood, adiposity, blood pressure

## Abstract

**Background:** Rapid infant weight gain is a risk factor for childhood obesity. This relationship may depend on whether infant weight gain is preceded by in-utero growth restriction.

**Aim**: Examine whether fetal growth modifies the relationship between infant weight gain and childhood adiposity and blood pressure.

**Subjects and methods:** 786 children in the Southampton Women’s Survey. We related infant weight gain (weight at 2 years-birth weight) to body mass index (BMI), %body fat, trunk fat (kg), systolic (SBP) and diastolic blood pressure (DBP) at age 6–7 years. Mean estimated fetal weight (EFW) between 19–34 weeks and change in EFW (19–34 weeks) were added to models as effect modifiers.

**Results:** Infant weight gain was positively associated with all childhood outcomes. We found no evidence that these effects were modified by fetal growth (*p* > .1 for all interaction terms). For example, a 1 standard deviation (SD) increase in infant weight gain was associated with an increase in BMI z-score of 0.51 (95% CI 0.37;0.64) when EFW-change was set at -2 SD-scores compared with an increase of 0.41 (95% CI 0.27;0.54, *p*_(interaction)_=.48) when set at 2 SD-scores.

**Conclusion:** The documented adverse consequences of rapid infant weight gain may occur regardless of whether growth was constrained in-utero.

## Introduction

Rapid infant weight gain is associated with increased weight in later life. A recent meta-analysis observed a four-fold increased odds of overweight/obesity (OR 4.16, 95% CI 3.26, 5.32) in childhood in those who previously demonstrated rapid infant weight gain (Zheng et al. 2018). Less is known about the relationship between infant weight gain and childhood cardiometabolic outcomes. A small number of studies have, however, observed positive associations between rapid infant weight gain and blood pressure (Huxley et al. 2000; Belfort et al. 2007; Singhal et al. 2007), glucose and insulin metabolism (Crowther et al. 1998; Ong et al. 2004), total-to-high density lipoprotein cholesterol (HDL) ratio (Bekkers et al. 2011), and triglycerides (Soto et al. 2003).

“Catch-up growth” during infancy has historically been determined based on linear growth. It has been considered a normal response to fetal constraint in-utero and part of the natural growth re-assortment that occurs in the first 1–2 years of life. For example, in the first 13 months of life, it has been observed that as many as two thirds of all infants shift centiles to achieve a new growth canal (Smith et al. 1976). More recently, focus has shifted to the epidemiology of “rapid infant weight gain”, with Ong et al reporting that more than 30% of infants display a change in weight-for-age SD score between 0–2 years >0.67 (Ong et al. 2000). If, as was originally thought for catch-up growth, rapid infant weight gain is a natural response to fetal constraint in-utero, it would not be expected to be associated with deleterious longer-term outcomes. However, the positive association observed between rapid infant weight gain and future adiposity and cardiometabolic risk factors has also been reported both in preterm and small-for-gestational-age infants (Corvalan et al. 2007; McCarthy et al. 2007; De Lucia Rolfe et al. 2010; Singhal 2017). This suggests that rapid weight gain in infancy, regardless of whether it is in response to a growth constraint in-utero, is deleterious for subsequent cardiometabolic health. A recent systematic review supports this view (Matthews et al. 2017); while a consistent positive association was observed between infant weight gain and subsequent obesity, 15 out of 18 eligible studies did not observe an interaction effect with birthweight. However, weight at birth is only a proxy for fetal growth and does not capture the patterns (e.g. fetal constraint) that lead to a given birthweight.

Three papers from the Generation R birth cohort study based in the Netherlands, have shown that the relationship between infant weight gain and childhood outcomes differs according to direct measures of fetal growth. In these studies, “fetal growth acceleration” and “fetal growth deceleration” were defined as a difference between 20 week estimated fetal weight (EFW) z-score and birthweight z-score > 0.67 and < −0.67, respectively. These were then related to infant weight gain (difference in weight-for-age z-scores between birth-6 months, 6–12 months and 12–24 months, with a difference of >0.67 and < −0.67 z-scores indicating “infant growth acceleration” and “deceleration”, respectively). While all three studies showed an interaction effect, the effects differed depending on the outcome, with some outcomes associated with “reduced fetal + increased infant” growth while others were associated with “increased fetal + increased infant” growth. As such, the results from these three studies are equivocal and further research in different cohorts is required.

The aim of the present study was to assess whether the relationship of infant weight gain (0–2 year) with childhood adiposity and blood pressure was modified by fetal growth in the Southampton Women’s Survey, a population-based cohort study in the United Kingdom.

## Subjects and methods

### Participants

We used data from the Southampton Women’s Survey, a prospective cohort study of 12,583 non-pregnant women aged 20–34 years recruited from the general population (Inskip et al. 2006). A total of 3,158 of these women were followed through a subsequent pregnancy and delivered a live-born singleton infant. The study had full approval from the Southampton and Southwest Hampshire Local Research Ethics Committee and all participants gave written informed consent.

Of the 3158 liveborn singleton infants, 3031 were targeted for 6-year follow-up and 2048 were visited at home at 6–7 years of age. Of these, 1614 were willing to attend a clinic for further measurements, and a subset of 1240 had a whole-body DXA scan due to willingness of the participant and availability of facilities. The analysis was limited to those with complete fetal growth, infant weight gain and 6-year BMI data (*n* = 786, Supplementary Figure 1).

### Exposure: infant weight gain (0–2 years)

Birth weight was measured using calibrated digital scales (Seca, UK). The 2-year assessment was performed by a research nurse during a home visit, in which weight was measured using calibrated digital scales (Seca Ltd, UK). Infant weight gain was calculated by subtracting birth weight from weight at 2 years.

### Outcomes: childhood adiposity and blood pressure (6–7 years)

Whole-body scans were obtained using a Hologic Discovery A instrument with APEX 3.0 software (Hologic, Bedford, MA). To encourage compliance, a sheet with appropriate pictures was laid on the couch, and to help reduce movement artefact, the children were shown a suitable DVD. The total radiation doses for the scan was 4.7 μSv. From this scan sex- and age-adjusted estimates of percent body fat and trunk fat (kg) were obtained. During the assessment, the child’s height (using a Leicester height measure, Seca) and weight (using calibrated digital scales, Seca) were also measured. At the clinic visit, a single measurement of seated blood pressure was obtained using a Dinamap monitor. The five outcome variables investigated were BMI, trunk fat, per cent body fat and systolic (SBP) and diastolic blood pressure (DBP).

### Effect modifier: fetal growth (19–34 weeks gestation)

Our two effect modifiers were the mean of, and difference between, the estimated fetal weights (EFW, grams) obtained at 19 and 34 weeks. These were calculated for males and females separately (Supplementary Table 1). More details about the measurement protocol for obtaining fetal parameters and the calculation of EFW can be found in the Supplementary material.

### Covariates

At 11 weeks’ gestation, women were interviewed by research nurses. At this visit women provided information on whether they were currently smoking, resulting in a binary smoking status variable (yes/no). Women were weighed again and using the heights recorded from the initial pre-pregnancy interview, first-trimester body mass index (BMI) was calculated. Data relating to pregnancy characteristics were extracted from obstetric notes. From these, a binary gestational diabetes variable was obtained (yes/no). The occupation of the woman and her partner were obtained, from which the social class was determined (according to the Registrar General classifications); the dominant social class of the woman and her partner was used for analysis. This resulted in a categorical variable with the following values: “professional”; “management and technical”; “skilled non-manual”; “skilled manual”; “partly skilled”; and “unskilled”.

### Statistical analysis

We used general linear regression to investigate how the association between infant weight gain (0–2 years) and each outcome was modified by mean EFW (19–34 weeks) and EFW change (19–34 weeks). In the first set of models, each outcome was regressed on infant weight gain. The second set of models included the interaction between mean EFW (19–34 weeks) and infant weight gain. The third set included the interaction between EFW change (19–34 weeks) and infant weight gain. 95% confidence intervals of the interaction terms were obtained, and we also performed a test of nested models, where the null hypothesis was that the added interaction term was equal to zero. Therefore, a significant result (*p* < .05) on this test suggests that the models including the interaction term(s) (i.e. the less restrictive models) are a better fit to the data.

Unadjusted analyses were conducted first, before adjusting for confounding variables (maternal first trimester BMI, maternal smoking during pregnancy, gestational diabetes, and household social class). These confounding variables were identified with the use of a directed acyclic graph (DAG) (Supplementary Figure 2).

Missing outcome and covariate data were handled using multiple imputation by chained equations (MICE)(Royston and White 2011), combining estimates using Rubin’s rules (Rubin 2004). We imputed back to the sample with complete exposure, modifier and 6-year BMI data (*n* = 786). In order to identify whether bias was introduced by limiting our analysis to those with complete exposure, modifier and outcome data, supplementary analyses comparing maternal and neonatal data of those in/excluded were performed (Supplementary Table 2).

For consistency across the two age periods, all effect sizes are presented per standard deviation (SD). To illustrate any interaction, we produced a plot for each outcome showing how the infant weight gain effect size varies across the distribution of each fetal growth variable.

### Supplementary analyses

Analyses were repeated using (1) infant weight gain 0–6 months (instead of 0–2 years), (2) infant weight gain 0–12 months (instead of 0–2 years), and (3) birth weight (instead of the fetal growth variables).

All analyses were conducted using Stata version 15 (Stata Corp, College Station, TX, USA).

## Results

The mean birth weight of the sample was 3470 g (SD = 552) (males: 3531 (538); females: 3408 (558)), with a median gestational age at birth of 40 weeks (IQR: 39.1,41.0). Mean weight at 2 years was 12.6 kg (SD = 1.4) (males: 12.9 (1.3); females: 12.3 (1.5)), with median weight gain between birth at 2 years of 9.1 kg (IQR: 8.2, 10.0) (males: 9.3 (8.6, 10.2); females: 8.8 (8.0, 9.6)). Further sample characteristics are reported in Table 1.

**Table 1.** Fetal, infant, and maternal characteristics (*n* = 786).

			% missing
Fetal characteristics			
Sex			
Males	*n* (%)	395 (50.3)	
Females	*n* (%)	391 (49.7)	
EFW at 19-week scan (g)	Mean (SD)	310.3 (39.2)	
Gestational age at 19-week scan (weeks)	Mean (SD)	19.5 (0.5)	
EFW at 34-week scan (g)	Mean (SD)	2483.1 (291.3)	
Gestational age at 34-week scan (weeks)	Mean (SD)	34.4 (0.5)	
Infant characteristics			
Birthweight (g): males	Mean (SD)	3531 (538)	
Birthweight (g): females	Mean (SD)	3408 (558)	
Gestational age at birth (weeks): males	Median (IQR)	40 (38.9,41.0)	
Gestational age at birth (weeks): females	Median (IQR)	40 (39.2, 41.0)	
Weight at 2-year visit (kg): males	Mean (SD)	12.9 (1.3)	
Weight at 2-year visit (kg): females	Mean (SD)	12.3 (1.5)	
Age at 2-year visit (years): males	Median (IQR)	2.0 (2.0, 2.1)	
Age at 2-year visit (years): females	Median (IQR)	2.0 (2.0, 2.1)	
Weight gain 0–2 years (kg): males	Median (IQR)	9.3 (8.6, 10.2)	
Weight gain 0–2 years (kg): females	Median (IQR)	8.8 (8.0, 9.6)	
Maternal characteristics			
Age at recruitment (years)	Mean (SD)	28.4 (3.8)	
Ethnicity (White)^a^	*n* (%)	761 (96.8)	
First trimester BMI (kg/m^2^)	Median (IQR)	24.9 (22.7, 28.4)	22.4
Gestational diabetes (yes)	*n* (%)	8 (1.0)	
Preeclampsia (yes)	*n* (%)	19 (2.4)	
Multiparity (yes)	*n* (%)	367 (46.7)	
Smoking in pregnancy (yes)^a^	*n* (%)	70 (9.1)	1.7
Alcohol in 1st trimester (units per week)^a^	Median (IQR)	0.3 (0, 1.5)	21.5
Educational level (≥university degree)^a^	*n* (%)	210 (26.8)	0.3
Household Social class^a^^b^			1.7
(Professional or Management)	*n* (%)	513 (65.3)	

^a^Based on self-report.

^b^According to Registrar General 1990 classification.

Most baseline differences between those included and excluded from the analysis were small, however, infants included in the sample were more likely to be born to older and more educated mothers, from higher social backgrounds and who were less likely to have smoked during pregnancy (Supplementary Table 2). All results presented are based on multiply imputed data, with little differences observed in the effect estimates when analyses were based on complete case data only (data not shown).

### BMI z-score

A 1 SD-score increase in infant weight gain was associated with a 0.46 (95% CI: 0.40, 0.52) increase in BMI z-score (Table 2).

**Table 2.** Relationship of infant weight gain between ages 0–2 years with adiposity and blood pressure outcomes at age 6–7 years.

	BMI z-score		Percent body fat		Trunk fat (kg)		Systolic blood pressure (mmHg)		Diastolic blood pressure (mmHg)	
	β	95% CI	β	95% CI	β	95% CI	β	95% CI	β	95% CI
Unadjusted										
Infant weight gain (0–2 years)^a^	0.50	0.44, 0.56	1.49	1.13, 1.85	0.41	0.35, 0.48	1.53	0.50, 2.56	0.54	−0.22, 1.31
Adjusted^b^										
Infant weight gain (0–2 years)^a^	0.46	0.40, 0.52	1.32	0.96, 1.67	0.38	0.32, 0.44	1.53	0.49, 2.57	0.53	−0.23, 1.29

^a^Estimates are presented per SD change.

^b^Adjusted for maternal first-trimester BMI, smoking in pregnancy, social class, and gestational diabetes.

**Figure 1.**
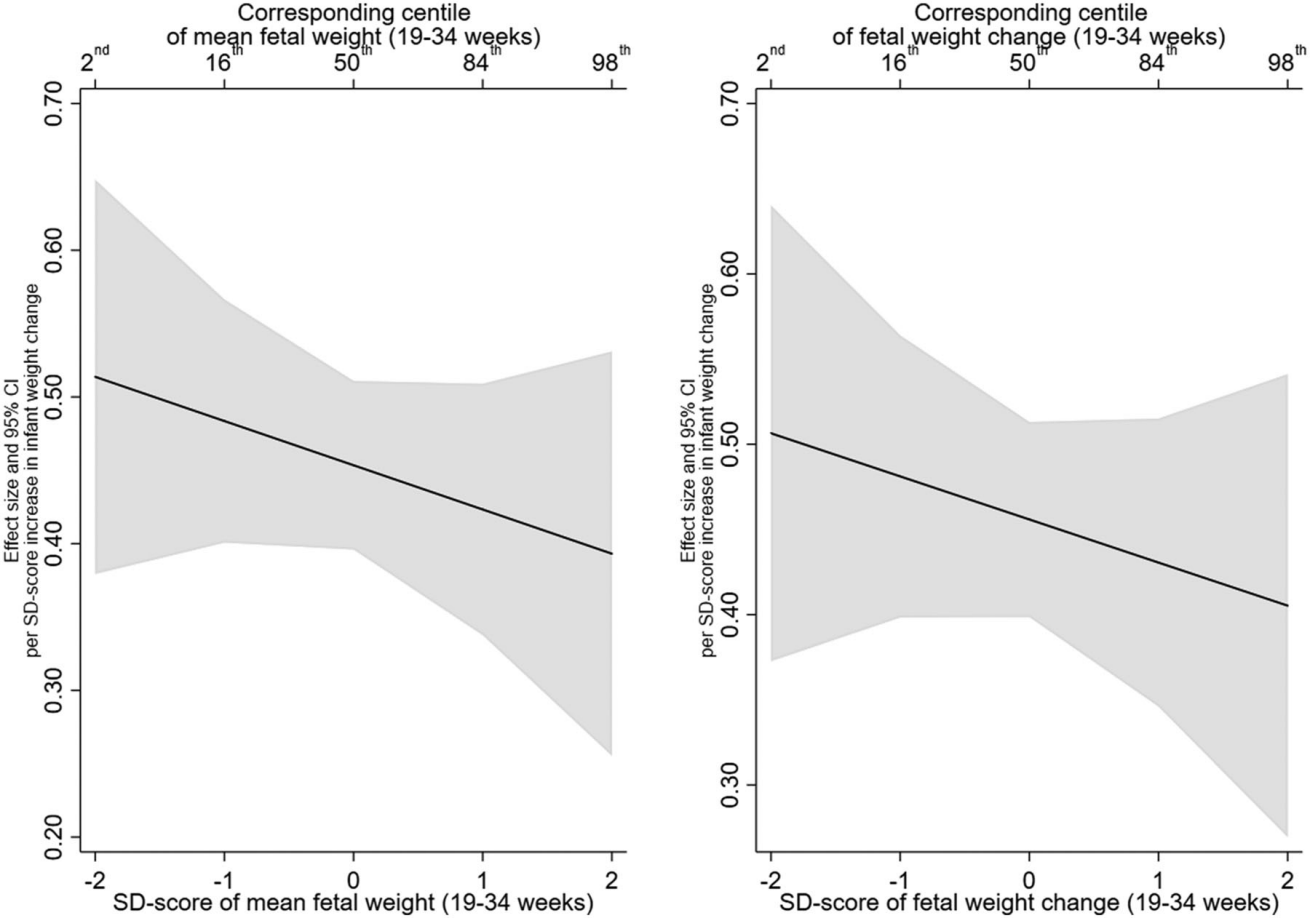
Estimated relationship of infant weight gain (0-2 years) with BMI z-score (6–7 years), across the distribution of mean fetal weight (19–34 weeks) and fetal weight change (19-34 weeks).

There was no evidence that this effect was modified by mean fetal weight or fetal weight change. For example, the infant weight gain X mean EFW (19–34 weeks) interaction term was −0.03 (95% CI:−0.09, 0.03, *p* = .39) and the infant weight gain X EFW change (19–34 weeks) was −0.02 (95% CI:−0.08, 0.04, *p* = .48) (Table 3 and Supplementary Table 3 for unadjusted estimates). In addition, testing whether the inclusion of the interaction terms resulted in better fitting models did not support their inclusion (minimum *p* > .35). Figure 1 illustrates the lack of interaction, with mean EFW (19–34 weeks) in the left panel and EFW change (19–40 weeks) in the right panel. The left panel shows that BMI z-score increases by 0.51 (95% CI: 0.38, 0.65) per SD increase in infant weight gain at a mean EFW SD-score of −2 (2nd centile), compared with an increase of 0.39 (95% CI: 0.26, 0.53) at a mean EFW SD-score of 2 (98th centile).

**Table 3.** Confounder-adjusted relationships of infant weight gain between ages 0–2 years with adiposity and blood pressure outcomes at age 6–7 years, testing for interactions with fetal weight variables.

	BMI z-score			Percent body fat			Trunk fat (kg)			Systolic blood pressure (mmHg)			Diastolic blood pressure (mmHg)		
	β	95% CI	*p*_(interaction)_	β	95% CI	*p*_(interaction)_	β	95% CI	*p*_(interaction)_	β	95% CI	*p*_(interaction)_	β	95% CI	*p*_(interaction)_
Model 1^a^															
Infant weight gain (0–2 years)^b^	0.45	0.40, 0.51	–	1.31	0.96, 1.67	–	0.38	0.32, 0.44	–	1.50	0.45, 2.54	–	0.53	−0.24, 1.29	–
Mean fetal weight (19–34 weeks)^b^	0.12	0.06, 0.18	–	−0.02	−0.39, 0.36	–	0.01	−0.05, 0.08	–	−0.03	−1.19, 1.14	–	−0.19	−0.96, 0.58	–
Infant weight gain X Mean fetal weight	−0.03	−0.09, 0.03	.39	−0.11	−0.48, 0.26	0.56	−0.04	−0.11, 0.03	0.29	−0.83	−1.80, 0.14	.09	−0.38	−1.10, 0.34	.30
Model 2^a^															
Infant weight gain (0–2 years)^b^	0.46	0.40, 0.51	–	1.31	0.96, 1.67	–	0.38	0.32, 0.44	–	1.50	0.46, 2,55	–	0.53	−0.23, 1.28	–
Change in fetal weight (19–34 weeks)^b^	0.13	0.07, 0.19	–	−0.01	−0.36, 0.38	–	0.02	−0.05, 0.09	–	−0.02	−1.16. 1.12	–	−0.17	−0.95, 0.61	–
Infant weight gain X Foetal weight change	−0.02	−0.08, 0.04	.48	−0.06	−0.43, 0.31	0.75	−0.02	−0.09, 0.05	0.52	−0.91	−1.89, 0.07	.07	−0.38	−1.10, 0.33	.29

^a^Adjusted for maternal first-trimester BMI, smoking in pregnancy, social class, and gestational diabetes.

^b^Estimates are presented per SD change.

### Per cent body fat and trunk fat (kg)

A 1 SD-score increase in infant weight gain was also positively associated with percent body fat (1.32; 95% CI: 0.96, 1.67) and trunk fat (0.38; 95% CI: 0.32, 0.44) (Table 2).

**Figure 2.**
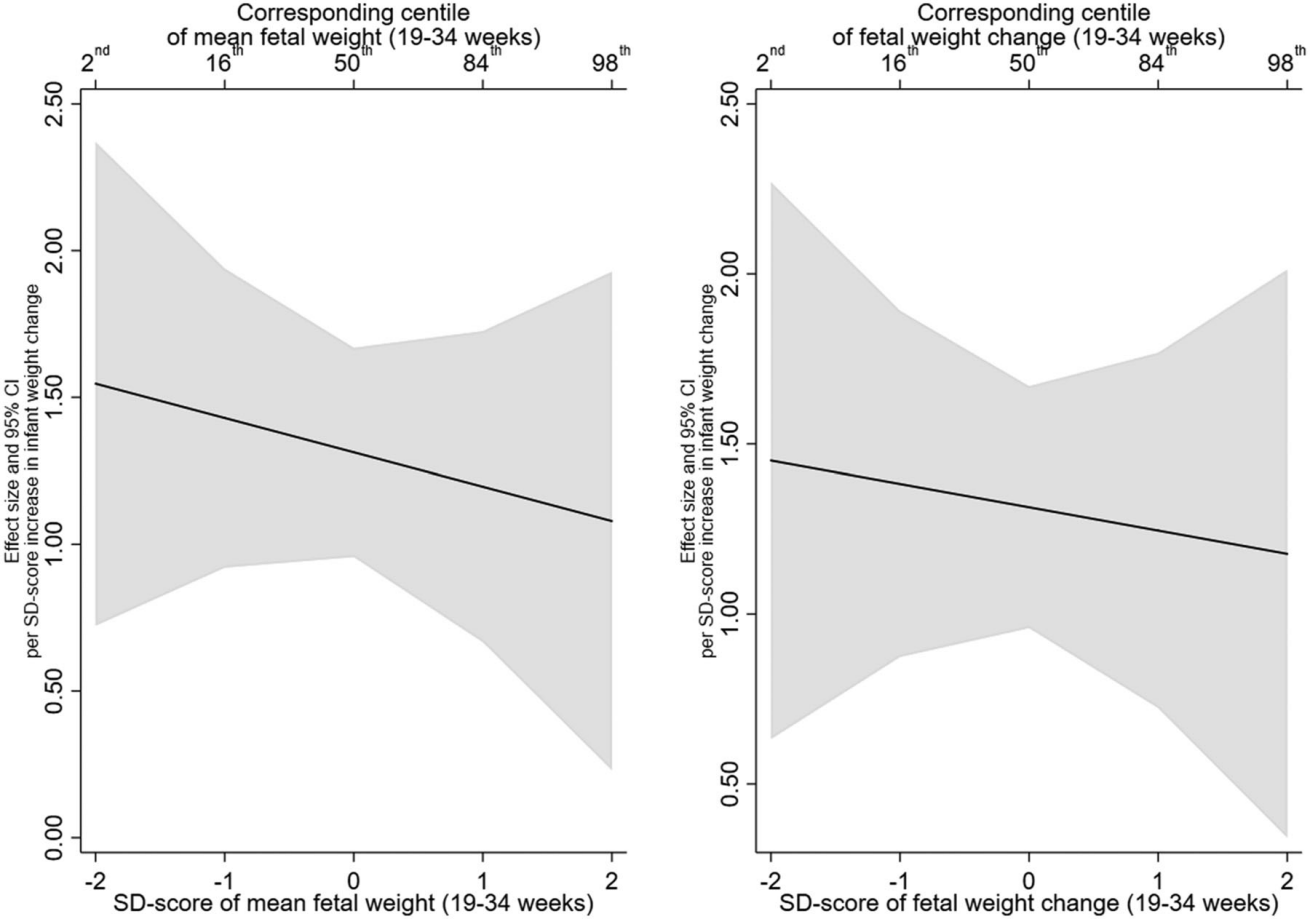
Estimated relationship of infant weight gain (0–2 years) with per cent body fat (6–7 years), across the distribution of mean fetal weight (19–34 weeks) and fetal weight change (19–34 weeks).

**Figure 3.**
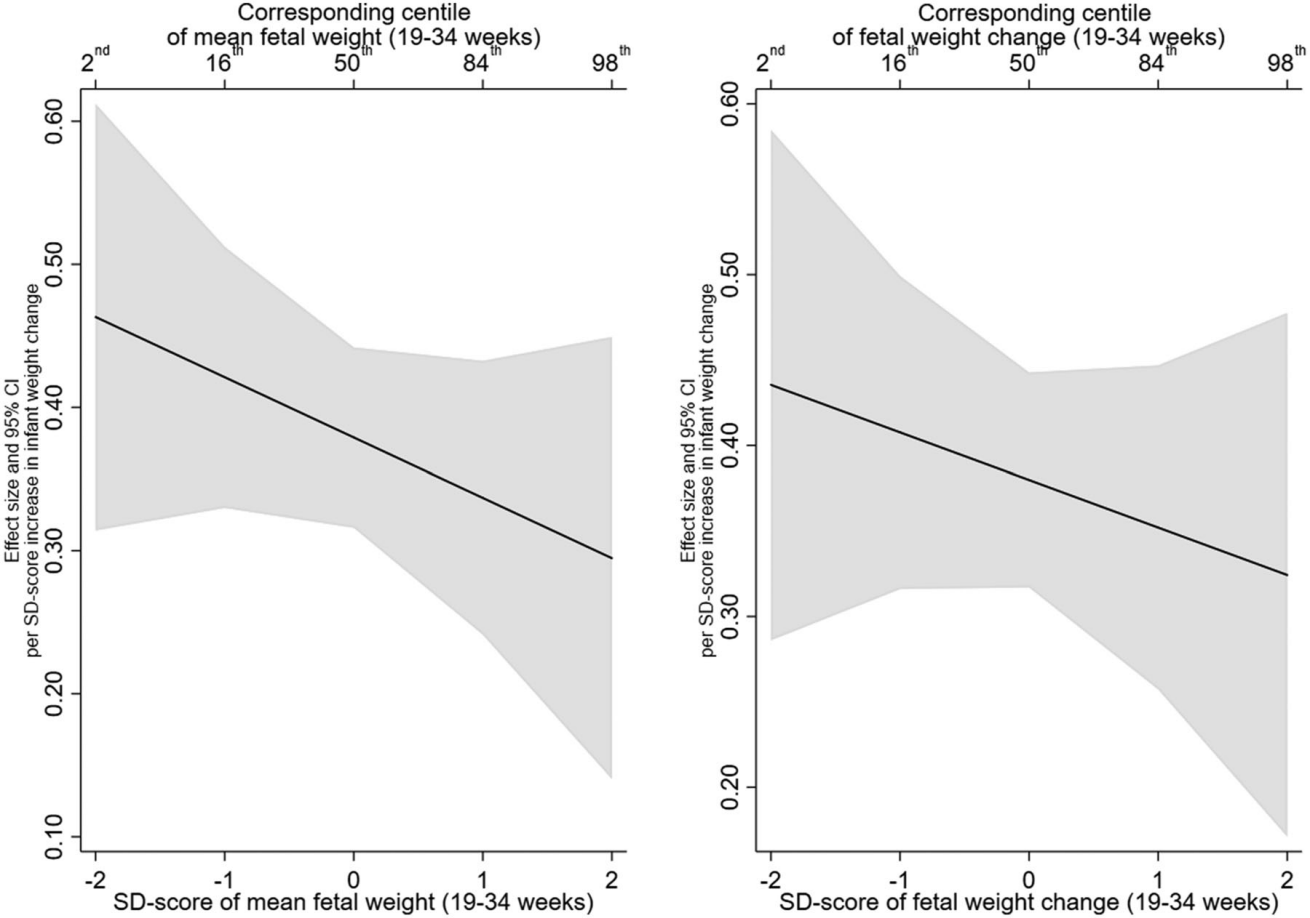
Estimated relationship of infant weight gain (0–2 years) with trunk fat (kg) (6–7 years), across the distribution of mean fetal weight (19–34 weeks) and fetal weight change (19–34 weeks).

There was no evidence that this effect was modified by mean EFW or EFW change, with all interaction effect sizes close to the null (Table 3). In addition, testing whether the inclusion of the interaction terms resulted in better fitting models did not support their inclusion (minimum *p* > .25). The lack of interaction with fetal growth variables can be further observed in the interaction plots for percent body fat and trunk fat, presented in Figures 2 and 3, respectively.

### SBP and DBP

A positive association was observed between infant weight gain and childhood systolic blood pressure, with a 1 SD-score increase in infant weight gain associated with a 1.53 mmHg (95% CI: 0.49, 2.57) increase in SBP. While a positive association was observed between a 1 SD-score increase in infant weight gain and DBP (β = 0.53), the 95% confidence intervals suggested an effect anywhere between −0.23 and 1.29.

**Figure 4.**
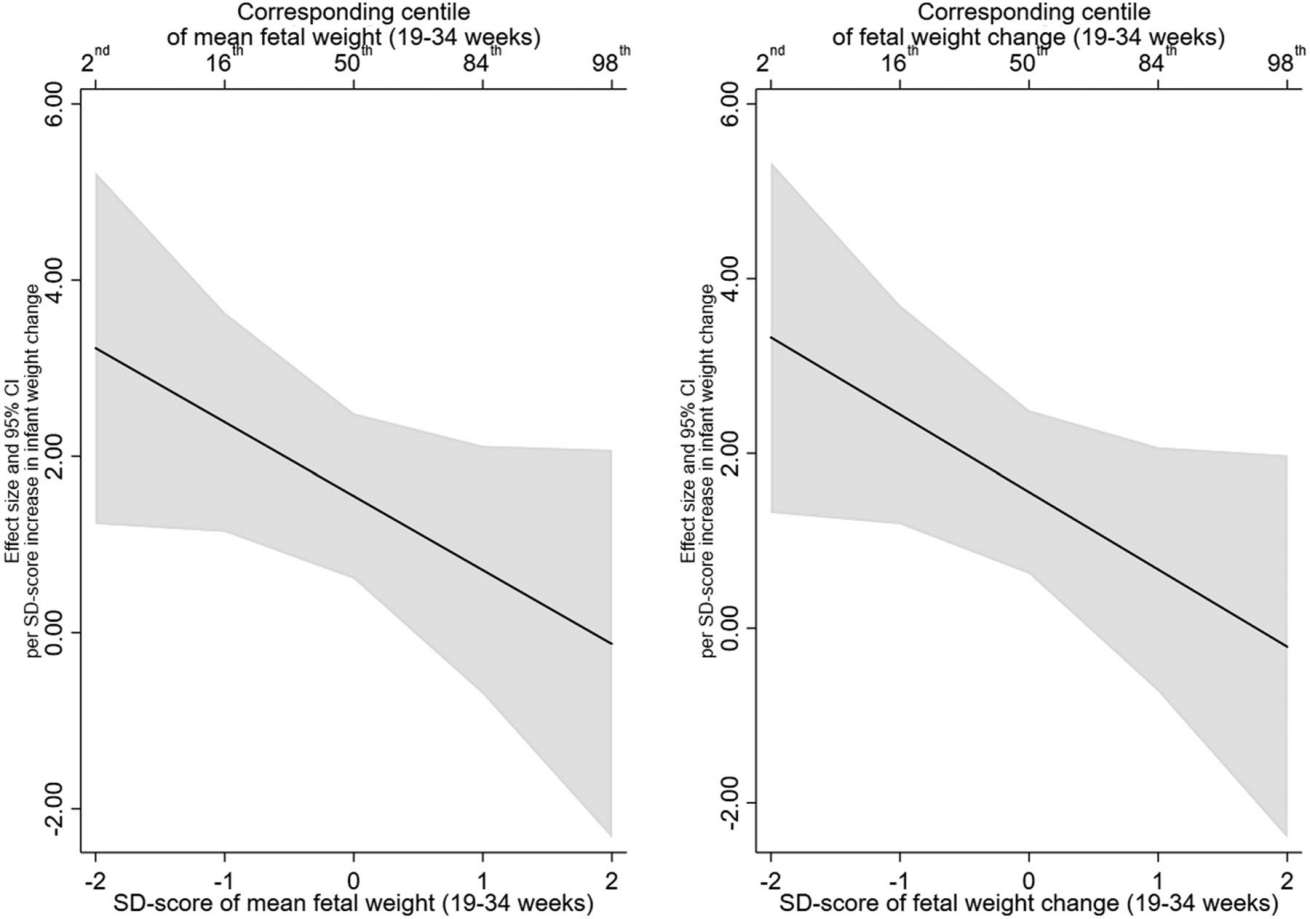
Estimated relationship of infant weight gain (0–2 years) with systolic blood pressure (mmHg) (6–7 years), across the distribution of mean foetal weight (19–34 weeks) and foetal weight change (19-34 weeks).

**Figure 5.**
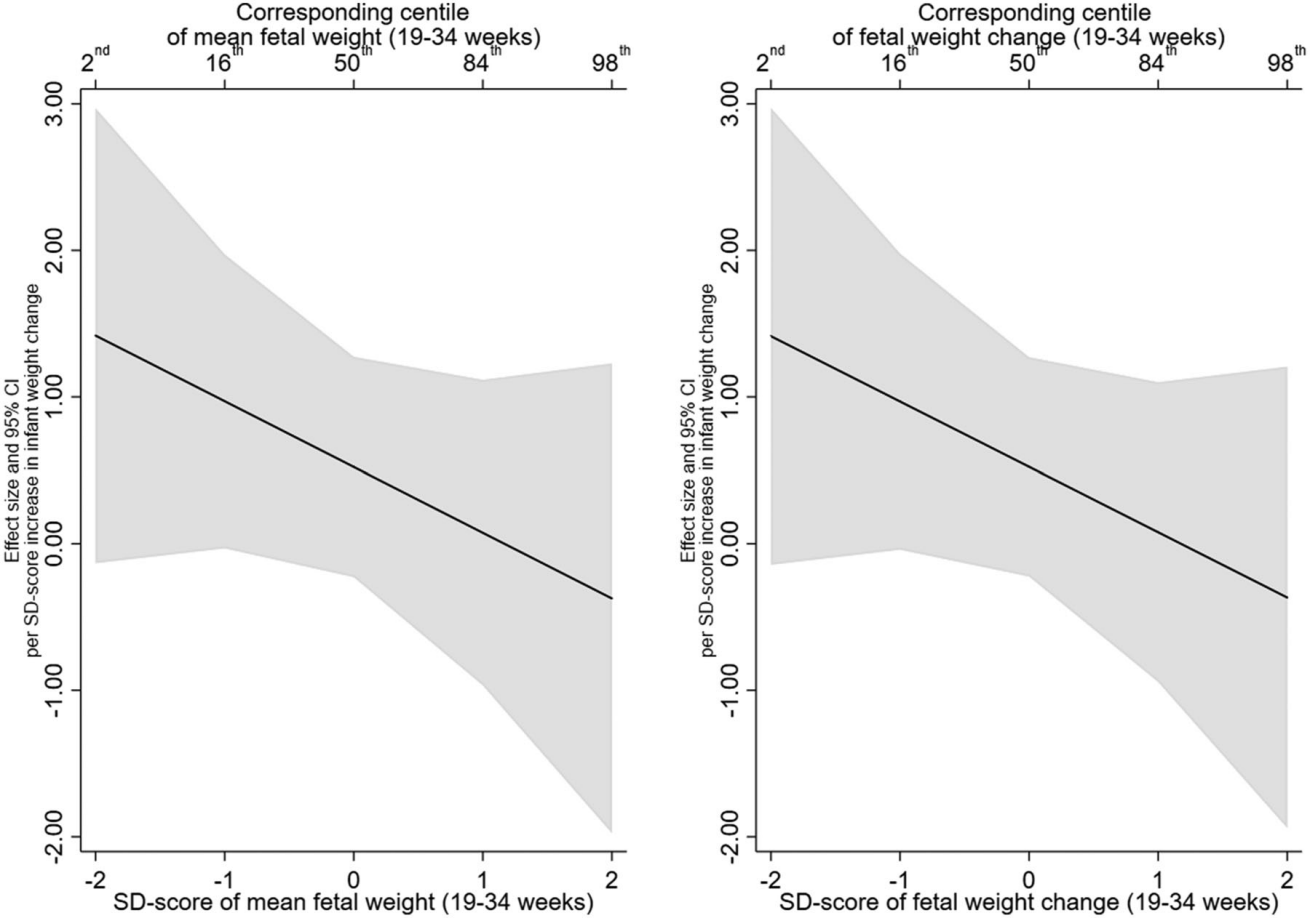
Estimated relationship of infant weight gain (0–2 years) with diastolic blood pressure (mmHg) (6–7 years), across the distribution of mean foetal weight (19–34 weeks) and foetal weight change (19–34 weeks).

For both SBP and DBP, there was no evidence that the association with infant weight gain was modified by mean EFW or EFW change (Table 3). Furthermore, the test of nested models did not provide evidence in support of the interaction models over the null model (minimum *p* > .1) and 95% confidence intervals were wide. Interaction plots for SBP and DBP are presented in Figures 4 and 5, respectively. For example, a 1 SD-score increase in infant weight gain was associated with an increase in SBP of 3.23 (95% CI: 1.23, 5.22) at a mean EFW SD-score of −2 reducing to −0.13 (95% CI: −2.33, 2.07) at a mean EFW SD-score of 2. Similarly, for the interaction between infant weight gain and EFW change, a 1 SD-score increase in infant weight gain was associated with an increase in SBP of 3.33 (95% CI: 1.32, 5.33) at an EFW change SD-score of −2 which reduced to -0.21 (95% CI: −2.40, 1.97) at an EFW change SD-score of 2.

### Supplementary analyses

Supplementary analyses using (1) infant weight gain 0–6 months (instead of 0–2 years), (2) infant weight gain 0–12 months (instead of 0–2 years), and (3) birth weight (instead of the fetal growth variables) found similar results (Supplementary Tables 4–6).

## Discussion

In the Southampton Women’s Survey, we observed positive associations between infant weight gain and childhood adiposity and blood pressure, as observed previously in many studies. We have added to these studies by investigating whether fetal growth modified these associations, and observed no evidence for a modifying role of growth in the second half of pregnancy.

The growth acceleration hypothesis (Singhal and Lucas 2004) suggests that any upward weight centile crossing in infancy, regardless of its cause (e.g. rapid infant weight gain following restricted growth in-utero or simply greater nutritional intake) will be associated with deleterious longer-term outcomes. The hypothesis was based on studies showing increased adverse cardiometabolic outcomes in those born preterm or small-for-gestational-age (SGA) (at term), who then subsequently demonstrated increased infant weight gain (Singhal 2017). However, the hypothesis would not be valid if rapid infant weight gain occurs mainly as a natural response to a period of fetal constraint. Size at birth is only a proxy for fetal growth and does not distinguish between the constitutionally small and the growth-restricted infant. Without such a distinction, it is not possible to establish whether periods of increased/reduced fetal growth modify the relationship between increased infant weight gain and cardiometabolic outcomes, as the hypothesis states. Our findings provide support for the growth acceleration hypothesis and suggest that increased weight gain in infancy, irrespective of EFW, may be positively associated with cardiometabolic outcomes in childhood.

These findings are further support for the decision to reorient the “fetal origins hypothesis” (Barker 1995), the notion that adverse exposures in-utero programme later sub-optimal health, to cover a greater period of early life. Indeed, as many growth-restricted fetuses display subsequent rapid infant growth, many of the observed associations between low birthweight and later cardiovascular risk could have simply been a proxy for the adverse effects of early postnatal weight gain.

Our findings differ from three studies from the “Generation R” cohort, which did observe interaction effects between infant weight gain and patterns of fetal weight gain (Gishti et al. 2014; Toemen et al. 2016; Vogelezang et al. 2019). However, across the three studies, the interaction effects differed depending on the outcome. For example, Gishti et al. (2014) observed that children with both “fetal growth acceleration” and “infant growth acceleration” had the highest body mass index (BMI), fat mass index, and abdominal fat at age 6 years. Conversely, children who had “fetal growth deceleration” and “infant growth acceleration” had the highest android/gynoid fat ratio and lowest lean mass index. In a second study (*n* = 6239), Toemen et al (2016) observed that children with “decelerated or normal fetal growth” followed by “accelerated infant growth” had higher blood pressure (compared to children with normal fetal and infant growth). Conversely, children who had “decelerated growth” during both gestation and infancy had a larger left ventricular mass (Toemen et al. 2016). Finally, in the third study, Vogelezang et al. (2019) observed that children who had either fetal growth deceleration or acceleration followed by accelerated infant growth displayed sub-optimal adiposity profiles in childhood. As such, the overall conclusion from these studies is unclear. These studies also categorised fetal and infant growth, which reduces power and classifies individuals who are close to, but on opposite sides of the z-score cut-point, as having very different, rather than very similar growth. We believe our approach of modelling fetal and infant growth as continuous variables provides a more powerful and realistic method of exploring their potential interaction.

In light of our findings, the postnatal nutritional management of those who experienced a sub-optimal fetal milieu (culminating in either preterm or term SGA birth), should be carefully considered. While the promotion of infant growth in length has been associated with favourable neurodevelopmental outcomes in those born preterm (Isaacs et al. 2009), it has also been observed that faster postnatal weight gain increases later risk factors for cardiovascular disease (CVD) in this group (Kerkhof et al. 2012; Ong et al. 2015). For those born SGA at term, evidence from high income countries suggests that greater infant weight gain increases later risk for obesity and CVD (Singhal 2016). In low-income countries, faster postnatal growth in length has been associated with lower morbidity in low birthweight infants (Jain and Singhal 2012), however it has also been shown that even transient rapid infancy weight gain (i.e. weight gain that is followed by growth faltering) is associated with greater adiposity in childhood and early adulthood (Salgin et al. 2015). Nonetheless, managing weight gain in infancy is far from straightforward and care must be taken in the leap from finding an association to deciding whether to intervene.

### Strengths

As well as BMI, we have investigated relationships with adiposity obtained via DXA, which is able to provide a more accurate reflection of whole body and regional adiposity. We have performed a number of supplementary analyses, including reparameterising infant weight gain and replacing fetal weight with birthweight. These analyses yielded similar associations to the main analysis and thus increase the robustness of our findings. The extensive data collection and number of variables available in SWS enabled us to adjust for all of the confounding variables identified in our DAG. We are, however, cautious not to refer to any association as “causal” as the possibility of the presence of residual confounding cannot be excluded and there is known error associated with the calculation of EFW (Milner and Arezina 2018). In participants with missing data, multiple imputation was used to impute missing values.

### Limitations

The use of EFW provided us with a measure of prenatal growth that could be used in conjunction with infant weight gain and also enabled some comparison to the related studies from the “Generation R” cohort (Gishti et al. 2014; Toemen et al. 2016; Vogelezang et al. 2019). The generation of EFW is, however, associated with error. In our study, EFW was generated according to the Hadlock formula (Hadlock et al. 1985). The most recent systematic review investigating the accuracy of different formulae for the calculation of EFW concluded that the Hadlock formula produced the most accurate results (Milner and Arezina 2018). While most baseline differences between those included and excluded from the analysis were small (Supplementary Table 2), our sample selected a more educated group of women from higher occupational social class backgrounds, which may limit the generalisability of our findings.

## Conclusions

The relationship of infant weight gain with cardiometabolic disease risk factors in childhood was not modified by fetal growth in the Southampton Women’s Study. This suggests that the documented adverse consequences of rapid infant weight gain may occur regardless of whether or not growth was constrained in-utero.

## Supplementary Material

Supplemental Material
