# Supplementary material for: Fetal growth does not modify the relationship of infant weight gain with childhood adiposity and blood pressure in the Southampton women’s survey
Source: Ann Hum Biol. 2020 May 20;47(2):150–8. doi: 10.1080/03014460.2020.1717616 (PMC7261399; doi:10.1080/03014460.2020.1717616)
Supplement: Supplemental Material [file IAHB_A_1717616_SM3942.pdf]

Supplementary material: *Fetal growth does not modify the relationship of infant weight gain with childhood adiposity and blood pressure in the Southampton Women's Survey*

Norris, T., Crozier, S.R., Cameron, N., Godfrey, K.M., Inskip, H. & Johnson, W

Supplementary figure 1:

Flow diagram for final analysis sample:

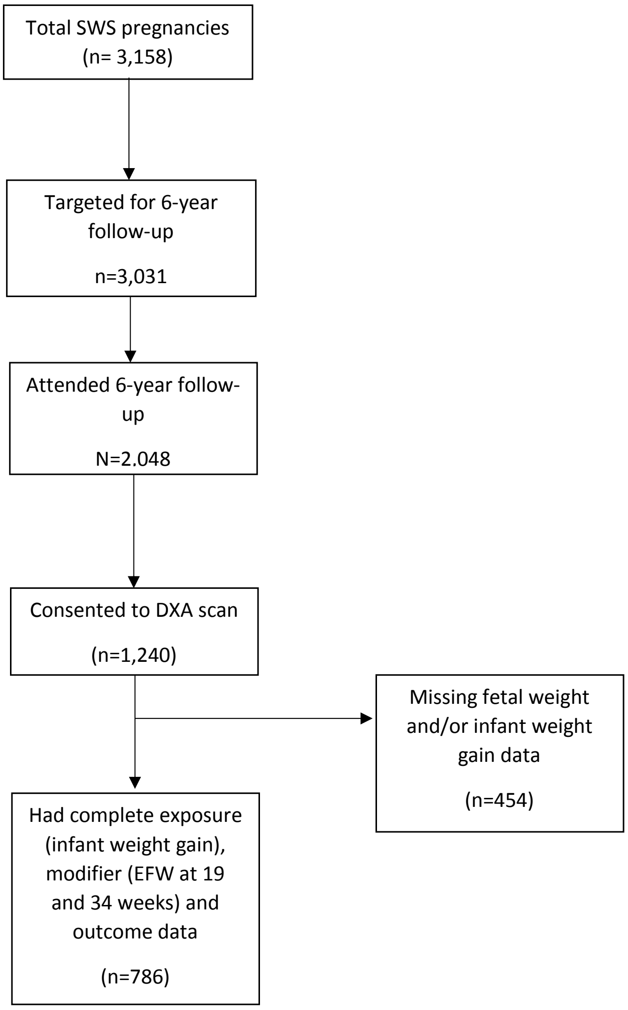

Modelling EFW trajectoriesFetal ultrasound data

Gestational age at birth was calculated from the date of the mother's last menstrual period (LMP) and confirmed by ultrasonography or calculated following an early dating scan if the mother had been uncertain about her LMP date. At 19- and 34-weeks' gestation, women underwent high-resolution ultrasound scanning using a Kretz Voluson 730 (GE Kretz Ultrasound, Tiefenbach, Austria) system or an Acuson Sequoia 512 (Siemens, Erlangen, Germany) system; the two systems were cross-calibrated. After establishing correct positioning according to standard anatomical landmarks, measurements of abdominal circumference (AC), head circumference (HC), and femur length (FL) were made on the frozen images using electronic calipers by the two operators, according to internationally accepted and validated methodology[1-3]. Each measurement was performed in triplicate, and the mean value was used for analysis. Using these parameters, we derived estimated fetal weight (EFW) using the Hadlock formula[4].

As there was no external reference for the creation of z-scores which covered the gestational period in this study and in order to address differences in age at ultrasound assessment, a decision was made to model fetal weight using linear mixed effects models, with child classed as level 2 and measurement occasion as level 1. Trajectories were based on EFW at 19- and 34-weeks' gestation, with birthweight also added, thus serving as a measure of weight at the end of the gestational period. We included a binary variable at level 1 to model the differences in error associated with the differences in measurement between EFW and birthweight. In an attempt to stabilise the increasing variability in fetal size with gestational age, modelling of EFW trajectories was done on the (natural) log-transformed scale, which has been advocated in previous studies of fetal growth [(5-8)]. As there was evidence of sex

~~differences in EFW trajectories, models were run separately for males and females. From these multilevel models, two random effects were obtained for each individual. These represent each individual's difference from the average predicted EFW at 19 weeks and difference from the average predicted weekly gain in EFW between 19 weeks and birth. These random effects were subsequently converted into z-scores so that the sizes of the coefficients were directly comparable.~~

Adjusting for confounding variables

The adjustment set was identified via literature searches and after holding consultative meetings with experts in the fields related to the exposure and outcome. Results of these searches/meetings were encapsulated in the form of directed acyclic graphs. The final DAG is included below and resulted in the adjustment for maternal BMI at start of pregnancy, maternal smoking during pregnancy, gestational diabetes, and social class.

Supplementary figure 2:

Formatted: Font: Bold

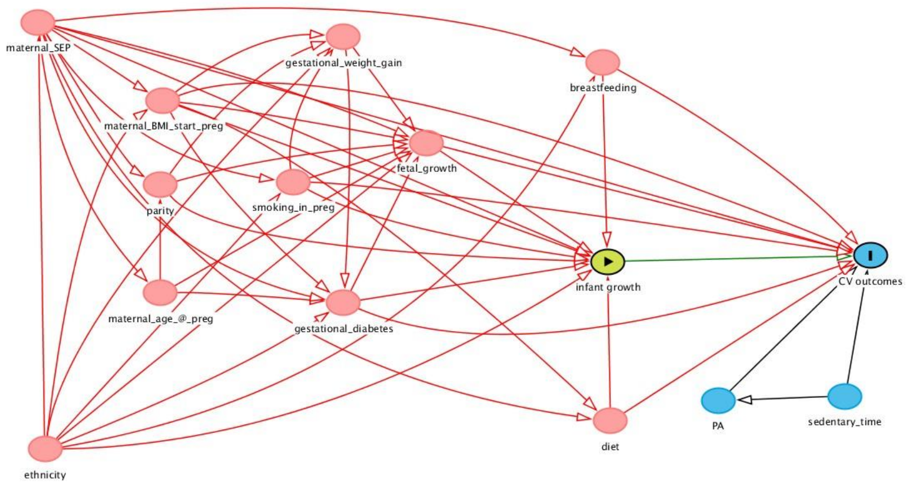

References:

1.Chitty LS, Altman DG, Henderson A, Campbell S. Charts of fetal size: 3. Abdominal measurements. BJOG: An International Journal of Obstetrics and Gynaecology 1994;101:125–131.

2. Chitty LS, Altman DG, Henderson A, Campbell S. Charts of fetal size: 2. Head measurements*. BJOG: An International Journal of Obstetrics and Gynaecology 1994;101:35–43.

3.Chitty LS, Altman DG, Henderson A, Campbell S. Charts of fetal size: 4. Femur length. British journal of obstetrics and gynaecology 1994;101:132–5.

4.Hadlock FP, Harrist RB, Sharman RS, Deter RL, Park SK. Estimation of fetal weight with the use of head, body, and femur measurements--a prospective study. American journal of obstetrics and gynecology 1985;151:333–7.

Supplementary table 1 Estimated fetal weight at 19 and 34 weeks, mean fetal weight and (19-34 weeks) and weight gain change between 19-34 weeks to birth based on multilevel models in 7868 babies in the Southampton Women's Survey

	Mean* ± SE	95% reference range
Females (n=392)		
Fetal weight at 19 weeks (g)	281.0 ± 1.8	277.5; 284.5
Weight gain 19 weeks–birth (% change/week)	14.9 ± 0.01	14.8; 15.0
Males (n=396)		
Fetal weight at 19 weeks (g)	292.9 ± 1.7	289.6; 296.4
Weight gain 19 weeks–birth (% change/week)	14.7 ± 0.01	14.6; 14.8

*coefficients are back-transformed from the (natural) log scale with weights at 19 weeks representing the geometric mean constant and the growth coefficients representing % change in weight per week.

	Mean (SD)
Females (n=391)	
Fetal weight (19 weeks) (g)	305 (39.3)
Gestational age at 19-week scan (weeks)	19.5 (0.6)
Fetal weight (34 weeks) (g)	2466 (294.8)
Gestational age at 34-week scan (weeks)	34.4 (0.5)
Mean fetal weight (19-34 weeks) (g)	1386 (156.8)
Fetal weight change (19-34 weeks) (g)	2161 (280.3)
Males (n=395)	
Fetal weight (19 weeks) (g)	315 (39.2)
Gestational age at 19-week scan (weeks)	19.5 (0.5)
Fetal weight (34 weeks) (g)	3000 (286.0)
Gestational age at 34-week scan (weeks)	34.5 (0.5)
Mean fetal weight (19-34 weeks) (g)	1407 (153.4)
Fetal weight change (19-34 weeks) (g)	2184 (269.5)

Formatted Table

Supplementary table 2 Characteristics of those in with complete exposure, modifier and outcome data (n=788) vs those excluded (n=2370)

	Complete exposure, modifier and outcome data (n=788)		Those missing either exposure, modifier or outcome data (n=2370)		*p* for diff*
	total n		*total n*		
Infant characteristics					
Sex (male, %)	788	396 (50)	2365	1237 (52)	p=0.32
Birthweight (g)	788	3470 (551)	2331	3418 (567)	p=0.03
Gestational age at birth (weeks)	788	40 (39; 41)	2337	40 (39; 41)	p=0.78
Maternal characteristics					
Age at recruitment (years)	788	28 (4)	2370	27 (4)	p<0.001
Ethnicity (White, %)	788	762 (97)	2369	2254 (95)	p=0.29
BMI in first trimester (kg)	611	25 (23; 28)	1541	25 (23; 29)	p=0.91
Education (degree, %)	786	211 (27)	2363	482 (20)	P<0.001
Occupational Social class (Professional or Management, n(%)))	788	514 (65)	1987	1157 (58)	p=0.001
Multiparity (yes, %)	788	369 (47)	2367	1174 (50)	p=0.18
Smoking during pregnancy (yes, %)	775	70 (9)	2215	415 (19)	p<0.001
Units of alcohol in 1st trimester (per week)	618	0.3 (0; 1.5)	1604	0.3 (0; 1.5)	p=0.65
Pregnancy characteristics					
Gestational diabetes (yes, %)	788	8 (1)	2368	30 (1)	p=0.58
Preeclampsia (yes, %)	788	19 (2)	2368	71 (3)	p=0.39

*Most baseline differences between those included and excluded from the analysis were small, however infants included in the sample were more likely to be born to older (28.4 years vs 27.4 years; *p* <0.001) and more educated mothers (% with degree: 276.8% vs 20.4%, *p*<0.001), from higher social backgrounds (% from ‘professional’ or ‘management’ social class: 65.2% vs 58.2%, *p*=0.001) and who were less likely to have smoked during pregnancy (9.0% vs 198.7%, *p* <0.001).

		Complete exposure, modifier and outcome data (n=786)		Those missing either exposure, modifier or outcome data (n=2372)	p for diff*
	total n		*total n*		
Infant characteristics					
Sex (male, %)	786	395 (50)	2367	1238 (52)	p=0.32
Birthweight (g)	786	3470 (552)	2333	3418 (567)	p=0.02
Gestational age at birth (weeks)	786	40 (39; 41)	2339	40 (39; 41)	p=0.75
Maternal characteristics					
Age at recruitment (years)	786	28 (4)	2372	27 (4)	p<0.001
Ethnicity (White, %)	786	762 (97)	2371	2254 (95)	p=0.25
BMI in first trimester (kg)	610	25 (23; 28)	1542	25 (23; 29)	p=0.93
Education (University degree, %)	784	210 (27)	2365	483 (20)	P<0.001
Occupational Social class (Professional or Management, n(%)))	786	513 (65)	1989	1158 (58)	p=0.001
Multiparity (yes, %)	786	367 (47)	2369	1176 (50)	p=0.15
Smoking during pregnancy (yes, %)	773	70 (9)	2217	415 (19)	p<0.001
Units of alcohol in 1st trimester (per week)	617	0.3 (0; 2)	1605	0.3 (0; 2)	p=0.64
Pregnancy characteristics					
Gestational diabetes (yes, %)	786	8 (1)	2370	30 (1)	p=0.58
Preeclampsia (yes, %)	786	19 (2)	2370	71 (3)	p=0.40

Supplementary Table 3. Unadjusted relationships of infant weight gain between ages 0-2 years with adiposity and blood pressure outcomes at age 6-7 years, testing for interactions with fetal weight variables

	BMI z-score		Percent body fat		Trunk fat (kg)		Systolic blood pressure (mmHg)		Diastolic blood pressure (mmHg)	
	β	95% CI	β	95% CI	β	95% CI	β	95% CI	β	95% CI
**Model 1**										
Infant weight gain (0-2 years)[a]	0.19	0.15, 0.23	0.58	0.34, 0.82	0.16	0.11, 0.20	0.52	-0.23, 1.27	0.49	-0.01, 0.99
Baseline fetal weight (19 weeks)[a]	0.20	0.15, 0.24	0.45	0.18, 0.71	0.14	0.09, 0.19	0.58	-0.13, 1.30	0.23	-0.30, 0.75
Infant weight gain X Baseline fetal weight	-0.01	-0.04, 0.01	-0.05	-0.20, 0.11	-0.01	-0.04, 0.02	-0.19	-0.76, 0.37	-0.16	-0.55, 0.23
**Model 2[b]**										
Infant weight gain (0-2 years)[a]	0.23	0.19, 0.28	0.69	0.44, 0.94	0.18	0.14, 0.23	0.74	-0.01, 1.49	0.52	0.02, 1.02
Fetal weight gain (19-40 weeks)[a]	0.18	0.12, 0.23	0.46	0.15, 0.77	0.10	0.05, 0.16	1.03	0.24, 1.82	0.29	-0.27, 0.85
Infant weight gain X Fetal weight gain	-0.01	-0.03, 0.02	-0.003	-0.14, 0.13	-0.004	-0.03, 0.02	-0.25	-0.69, 0.19	-0.28	-0.60, 0.05

[a]Estimates are presented per 0.67 z-scoreSD change, which is equivalent to one major centile band.
[b]Adjusted for baseline fetal weight (19 weeks).

	BMI z-score		Percent body fat		Trunk fat (kg)		Systolic blood pressure (mmHg)		Diastolic blood pressure (mmHg)	
	β	95% CI	β	95% CI	β	95% CI	β	95% CI	β	95% CI
**Model 1**										
Infant weight gain (0-2 years)[a]	0.48	0.42, 0.54	1.45	1.09, 1.81	0.41	0.34, 0.47	1.48	0.44, 2.52	0.54	-0.23, 1.30
Mean fetal weight (19-34 weeks)[a]	0.17	0.11, 0.23	0.33	-0.04, 0.69	0.08	0.02, 0.15	0.08	-1.05, 1.20	-0.18	-0.93, 0.56
Infant weight gain X Mean fetal weight	-0.04	-0.11, 0.02	-0.20	-0.58, 0.19	-0.06	-0.13, 0.02	-0.89	-1.85, 0.06	-0.40	-1.13, 0.33
**Model 2[b]**										
Infant weight gain (0-2 years)[a]	0.49	0.43, 0.55	1.46	1.10, 1.82	0.41	0.34, 0.47	1.49	0.46, 2.53	0.54	-0.22, 1.30
Change in fetal weight (19-34 weeks)[a]	0.18	0.12, 0.24	0.35	-0.02, 0.72	0.09	0.02, 0.15	0.09	-1.02, 1.19	-0.16	-0.92, 0.59
Infant weight gain X Fetal weight change	-0.4	-0.10, 0.02	-0.15	-0.53, 0.24	-0.04	-0.11, 0.03	-0.97	-1.93, -0.00	-0.40	-1.13, 0.32

Supplementary Table 4. Confounder-adjusted relationships of infant weight gain between ages 0-2 years with adiposity and blood pressure outcomes at age 6-7 years, testing for interactions with birth weight

	~~BMI z-score~~		~~Percent body fat~~		~~Trunk fat (kg)~~		~~Systolic blood pressure (mmHg)~~		~~Diastolic blood pressure (mmHg)~~	
	~~β~~	~~95% CI~~	~~β~~	~~95% CI~~	~~β~~	~~95% CI~~	~~β~~	~~95% CI~~	~~β~~	~~95% CI~~
~~**Model 1[a]**~~										
~~Infant weight gain (0-2 years)[b]~~	~~0.35~~	~~0.31, 0.40~~	~~1.00~~	~~0.72, 1.28~~	~~0.29~~	~~0.24, 0.34~~	~~1.19~~	~~0.37, 2.02~~	~~0.48~~	~~-0.06, 1.03~~
~~Birth weight[b]~~	~~0.38~~	~~0.33, 0.43~~	~~0.84~~	~~0.52, 1.16~~	~~0.27~~	~~0.22, 0.33~~	~~1.38~~	~~0.45, 2.31~~	~~0.06~~	~~-0.57, 0.70~~
~~Infant weight gain X Birth weight~~	~~0.004~~	~~-0.01, 0.02~~	~~0.02~~	~~-0.09, 0.13~~	~~0.003~~	~~-0.02, 0.02~~	~~-0.09~~	~~-0.54, 0.37~~	~~-0.19~~	~~-0.55, 0.16~~

[a]Adjusted for maternal first-trimester weight, smoking in pregnancy, social class, and gestational diabetes.
[b]Estimates are presented per ~~0.67 z-score~~SD change~~, which is equivalent to one major centile band~~.

	BMI z-score		Percent body fat		Trunk fat (kg)		Systolic blood pressure (mmHg)		Diastolic blood pressure (mmHg)	
	β	95% CI	β	95% CI	β	95% CI	β	95% CI	β	95% CI
**Model 1[a]**										
Infant weight gain (0-2 years)[b]	0.47	0.41, 0.53	1.33	0.97, 1.68	0.39	0.32, 0.45	1.58	0.55, 2.61	0.53	-0.23, 1.29
Birth weight[b]	0.20	0.14, 0.26	0.33	-0.03, 0.70	0.12	0.05, 0.18	0.55	-0.52, 1.61	-0.11	-0.88, 0.66
Infant weight gain X Birth weight	-0.03	-0.09, 0.02	0.12	-0.24, 0.47	0.01	-0.06, 0.07	-1.00	-2.23, 0.24	-0.20	-1.02, 0.61

Supplementary Table 5. Confounder-adjusted relationships of infant weight gain between ages 0-6 months with adiposity and blood pressure outcomes at age 6-7 years, testing for interactions with fetal weight variables

	BMI z-score		Percent body fat		Trunk fat (kg)		Systolic blood pressure (mmHg)		Diastolic blood pressure (mmHg)	
	β	95% CI	β	95% CI	β	95% CI	β	95% CI	β	95% CI
**Model 1**[a]										
Infant weight gain (0-6 months)[b]	0.14	0.09, 0.18	0.56	0.31, 0.81	0.11	0.07, 0.16	0.63	-0.12, 1.38	0.45	-0.03, 0.93
Baseline fetal weight (19 weeks)[b]	0.13	0.09, 0.18	0.19	-0.06, 0.44	0.08	0.03, 0.12	0.45	-0.27, 1.17	0.12	-0.39, 0.63
Infant weight gain X Baseline fetal weight	-0.01	-0.04, 0.02	-0.10	-0.27, 0.06	-0.01	-0.04, 0.02	-0.11	-0.66, 0.44	-0.17	-0.57, 0.23
**Model 2**[c]										
Infant weight gain (0-6 months)[b]	0.16	0.11, 0.20	0.61	0.35, 0.86	0.12	0.07, 0.17	0.78	0.04, 1.52	0.46	-0.02, 0.95
Fetal weight gain (19-40 weeks)[b]	0.12	0.06, 0.17	0.26	-0.04, 0.55	0.04	-0.01, 0.10	0.85	0.08, 1.62	0.13	-0.43, 0.68
Infant weight gain X Fetal weight gain	-0.01	-0.02, 0.02	-0.01	-0.14, 0.12	-0.002	-0.03, 0.02	-0.15	-0.55, 0.25	-0.23	-0.53, 0.08

[a]Adjusted for maternal first-trimester weight, smoking in pregnancy, social class, and gestational diabetes.
[b]Estimates are presented per 0.67 z-scoreSD change, which is equivalent to one major centile band.
[c]Adjusted for baseline fetal weight (19 weeks) and maternal first-trimester weight, smoking in pregnancy, social class, and gestational diabetes.

	BMI z-score		Percent body fat		Trunk fat (kg)		Systolic blood pressure (mmHg)		Diastolic blood pressure (mmHg)	
	β	95% CI	β	95% CI	β	95% CI	β	95% CI	β	95% CI
**Model 1[a]**										
Infant weight gain (0-6 months)[b]	0.29	0.23, 0.36	1.10	0.74, 1.48	0.24	0.17, 0.31	1.49	0.51, 2.47	0.57	-0.20, 1.33
Mean fetal weight (19-34 weeks)[b]	0.14	0.07, 0.21	0.05	-0.34, 0.43	0.03	-0.04, 0.10	0.05	-1.12, 1.22	-0.17	-0.94, 0.59
Infant weight gain X Mean fetal weight	-0.03	-0.09, 0.03	-0.10	-0.45, 0.25	-0.01	-0.08, 0.05	-0.51	-1.35, 0.34	-0.31	-0.94, 0.32
**Model 2**										
Infant weight gain (0-6 months)[b]	0.30	0.23, 0.36	1.10	0.73, 1.47	0.24	0.17, 0.31	1.49	0.52, 2.47	0.56	-0.20, 1.33
Change in fetal weight (19-34 weeks)[b]	0.15	0.09, 0.22	0.07	-0.31, 0.45	0.04	-0.04, 0.11	0.06	-1.09, 1.21	-0.14	-0.92, 0.64
Infant weight gain X Fetal weight change	-0.02	-0.08, 0.03	-0.04	-0.38, 0.29	-0.00	-0.07, 0.06	-0.53	-1.33, 0.28	-0.27	-0.88, 0.33

Supplementary Table 6. Confounder-adjusted relationships of infant weight gain between ages 0-12 months with adiposity and blood pressure outcomes at age 6-7 years, testing for interactions with fetal weight variables

	BMI z-score		Percent body fat		Trunk fat (kg)		Systolic blood pressure (mmHg)		Diastolic blood pressure (mmHg)	
	β	95% CI	β	95% CI	β	95% CI	β	95% CI	β	95% CI
**Model 1[a]**										
Infant weight gain (0-12 months)[b]	0.14	0.10, 0.18	0.54	0.31, 0.78	0.12	0.08, -0.17	0.51	-0.23, 1.25	0.42	-0.06, 0.90
Baseline fetal weight (19 weeks)[b]	0.14	0.10, 0.19	0.22	-0.05, 0.48	0.08	0.03, 0.13	0.50	-0.26, 1.25	0.21	-0.33, 0.75
Infant weight gain X Baseline fetal weight	-0.01	-0.04, 0.02	-0.03	-0.19, 0.13	-0.0001	-0.03, 0.03	-0.15	-0.74, 0.44	-0.21	-0.62, 0.20
**Model 2[c]**										
Infant weight gain (0-12 months)[b]	0.17	0.13, 0.21	0.60	0.36, 0.85	0.13	0.09, 0.18	0.68	-0.07, 1.43	0.43	-0.05, 0.91
Fetal weight gain (19-40 weeks)[b]	0.13	0.07, 0.18	0.27	-0.04, 0.58	0.05	-0.004, 0.11	0.97	0.14, 1.80	0.28	-0.31, 0.86
Infant weight gain X Fetal weight gain	-0.01	-0.03, 0.02	0.01	-0.12, 0.14	-0.0001	-0.02, 0.02	-0.22	-0.65, 0.21	-0.28	-0.60, 0.04

[a]Adjusted for maternal first-trimester weight, smoking in pregnancy, social class, and gestational diabetes.
[b]Estimates are presented per ~~0.67 z-score~~SD change~~, which is equivalent to one major centile band~~.
~~[c]Adjusted for baseline fetal weight (19 weeks) and maternal first-trimester weight, smoking in pregnancy, social class, and gestational diabetes.~~

	BMI z-score		Percent body fat		Trunk fat (kg)		Systolic blood pressure (mmHg)		Diastolic blood pressure (mmHg)	
	β	95% CI	β	95% CI	β	95% CI	β	95% CI	β	95% CI
**Model 1[a]**										
Infant weight gain (0-12 months)[b]	0.34	0.28, 0.40	1.20	0.84, 1.56	0.28	0.22, 0.35	1.47	0.41, 2.53	0.48	-0.33, 1.29
Mean fetal weight (19 weeks)[b]	0.13	0.06, 0.19	0.01	-0.37, 0.39	0.02	-0.05, 0.09	-0.02	-1.18, 1.15	-0.20	-0.97, 0.57
Infant weight gain X Mean fetal weight	-0.04	-0.10, 0.02	-0.00	-0.37, 0.39	-0.00	-0.07, 0.07	-0.68	-1.62, 0.25	-0.45	-1.14, 0.23
**Model 2**										
Infant weight gain (0-12 months)[b]	0.34	0.28, 0.40	1.20	0.83, 1.56	0.28	0.22, 0.35	1.48	0.42, 2.54	0.48	-0.33, 1.29
Change in fetal weight (19-34 weeks)[b]	0.14	0.08, 0.21	0.04	-0.34, 0.42	0.03	-0.04, 0.10	0.00	-1.14, 1.14	-0.17	-0.95, 0.62
Infant weight gain X Fetal weight change	-0.03	-0.10, 0.03	0.03	-0.33, 0.39	0.01	-0.06, 0.07	-0.75	-1.67, 0.17	-0.40	-1.10, 0.27
